# Prospective validation of the prognostic 31‐gene expression profiling test in primary cutaneous melanoma

**DOI:** 10.1002/cam4.2128

**Published:** 2019-04-05

**Authors:** Jennifer Keller, Theresa L. Schwartz, Jason M. Lizalek, Ea‐sle Chang, Ashaki D. Patel, Maria Y. Hurley, Eddy C. Hsueh

**Affiliations:** ^1^ Department of Surgery Saint Louis University St. Louis Missouri; ^2^ Department of Dermatology Saint Louis University St. Louis Missouri

**Keywords:** cutaneous melanoma, gene expression profile, prognosis

## Abstract

**Background:**

Gene expression profiling (GEP) has been integrated into cancer treatment decision‐making in multiple neoplasms. We prospectively evaluated the prognostic utility of the 31‐GEP test (DecisionDx‐Melanoma, Castle Biosciences, Inc) in cutaneous melanoma (CM) patients undergoing sentinel node biopsy (SNB).

**Methods:**

One hundred fifty‐nine patients (age 26‐88) diagnosed with melanoma between 01/2013 and 8/2015 underwent SNB and concurrent GEP testing. GEP results were reported as low‐risk Class 1 (subclasses 1A and 1B) or high‐risk Class 2 (subclasses 2A and 2B). Statistical analyses were performed with chi‐square analysis, *t* tests, log‐rank tests, and Cox proportional hazard models. Recurrence‐free survival (RFS) and distant metastasis‐free survival (DMFS) were estimated using Kaplan‐Meier method.

**Results:**

Median follow‐up was 44.9 months for event‐free cases. Median Breslow thickness was 1.4 mm (0.2‐15.0 mm). There were 117 Class 1 and 42 Class 2 patients. Gender, age, Breslow thickness, ulceration, SNB positivity, and AJCC stage were significantly associated with GEP classification (*P < *0.05 for all). Recurrence and distant metastasis rates were 5% and 1% for Class 1 patients compared with 55% and 36% for Class 2 patients. Sensitivities of Class 2 and SNB for recurrence were 79% and 34%, respectively. Of 10 SNB‐positive/Class 2 patients, 9 recurred. By multivariate analysis, only SNB result and GEP class were statistically associated with both RFS (*P* = 0.008 and 0.0001) and DMFS (*P* = 0.019 and 0.001).

**Conclusions:**

Gene expression profiling Class 2 result and SNB positivity were independently associated with recurrence and distant metastasis in primary CM patients. GEP testing may have additive prognostic utility in initial staging work‐up of these patients.

## 1 INTRODUCTION

The incidence of melanoma is increasing. For 2018, the number of new cases of melanoma is estimated to reach more than 87,000 and the number of melanoma‐related deaths will be more than 9,000.^1^ The standard for prognostication of melanoma patients is the American Joint Committee on Cancer's (AJCC) staging system. It is based on clinical and histopathologic variables such as Breslow thickness, ulceration, nodal involvement, in transit or satellite deposit, and presence of distant metastases.^2^, ^3^, ^4^ While each successive revision of the staging system has resulted in a more accurate reflection of patient prognosis, significant variance in survival still exists within each stage under the current AJCC classification.

Gerami et al first reported a prognostic gene expression profiling (GEP) test utilizing the 31‐gene panel for use in patients with cutaneous melanoma (CM).^5^ This GEP test is comprised of 28 discriminating genes and 3 control genes, which are evaluated using formalin‐fixed paraffin‐embedded tissue from the primary melanoma tumor. The test reports results as Class 1 (low risk, with subclasses 1A and 1B) and Class 2 (high risk, with subclasses 2A and 2B), which are derived from comparison of the gene expression from the tested tumor to a training set of 164 melanoma cases with known long‐term clinical outcomes.^5^ Since the initial development and validation study, several retrospective and prospective studies have demonstrated that GEP classification is significantly correlated with recurrence‐free survival (RFS), distant metastasis‐free survival (DMFS), overall, and melanoma‐specific survival.^6^, ^7^, ^8^, ^9^, ^10^

To further evaluate the prognostic utility of the GEP test, we conducted a prospective observational study in patients undergoing wide excision and sentinel node biopsy (SNB) for treatment of their primary CM at our institution. The GEP test was performed at the time of SNB. Patients were followed at regular intervals. We observed that GEP classification and SNB result were associated with both RFS and DMFS by multivariate analysis. The use of GEP in combination with current AJCC classification may further improve the prognostication schema for patients with primary CM.

## 2 MATERIALS AND METHODS

### 2.1 Patient population

One hundred seventy‐four clinically node‐negative patients diagnosed with CM between January 2013 and August 2015 opted for GEP testing and underwent SNB and wide excision of their primary tumor at the Department of Surgery, Saint Louis University, St. Louis, Missouri. Informed consent was obtained under an Institutional Review Board‐approved protocol. SNB was performed with same day pre‐operative lymphoscintigram with Tc‐99 tagged radioactive tracer followed by intraoperative lymphatic mapping with Lymphazurin blue dye. GEP testing was prospectively performed by Castle Biosciences, Inc, Friendswood, Texas, as previously described.^5^, ^6^, ^10^ Fifteen patients had insufficient tumor for GEP testing. The final study cohort was comprised of 159 patients with both GEP and SNB results.

### 2.2 Follow‐up

Patient follow‐up was performed as described below. AJCC stage I and II patients underwent physical exam (PE), chest X‐ray (AP and lateral views), and laboratory evaluation with complete blood count and complete metabolic panel every 6 months for the first 2 years then yearly for the subsequent 3 years. Stage III patients underwent baseline brain MRI and PET/CT or CT of chest/abdomen/pelvis (CT/CAP) followed by completion node dissection for SNB‐positive patients and subsequently followed every 3 months with PE, MRI of brain, CT C/A/P, or PET/CT and laboratory evaluation for the first year, then every 4 months for the second year, and every 6 months for the subsequent 3 years. All stage III patients were referred to Medical Oncology for discussion regarding adjuvant therapy. Patient information was prospectively entered into a secured database.

### 2.3 Statistical analysis

Comparisons between GEP class and covariates were made using chi‐square tests for categorical variables and *t* tests for continuous variables. Survival outcomes were defined as the time between date of diagnosis and date of disease recurrence (RFS) or distant metastasis (DMFS) or last follow‐up. Survival curves were estimated by the nonparametric Kaplan‐Meier method. Log‐rank tests were used for univariate analysis of categorical variables to determine differences between curves. Univariate analysis of continuous variables was performed using the Cox proportional hazard regression method and all statistically significant variables are reported. Multivariate Cox proportional hazards models including all variables significant in univariate analyses were used to examine the association of GEP class with RFS or DMFS. *P* value < 0.05 was considered significant. All statistical analyses were two‐tailed.

## 3 RESULTS

### 3.1 Patient demographics

For the cohort with SNB and GEP results (n = 159; Table 1), the median Breslow thickness was 1.4 mm (range: 0.2‐15.0 mm). The median age was 59 (range: 26‐88) and the majority (61.6%) of patients were male. Thirty‐eight patients (24%) had ulceration of their primary tumors. Distribution of primary site was 16.4% head and neck (n = 26), 40.9% extremity (n = 65), and 42.8% truncal location (n = 68). SNB was positive for metastatic melanoma in 20 patients (12.6%). Three patients had satellite deposits in wide excision specimen with negative SNB. Thus, 23 patients had pathologic AJCC stage III melanoma following surgery. There were 117 Class 1 (91 subclass 1A and 26 subclass 1B) and 42 Class 2 patients (12 subclass 2A and 30 subclass 2B). GEP classification was significantly associated with gender, age, ulceration, Breslow thickness, SNB positivity, and AJCC stage (*P* = 0.009, 0.0001, <0.0001, <0.0001, 0.011, and <0.0001, respectively; Table 1).

**Table 1:** Patient demographics

	GEP^a^ Class 1 (n = 117)	GEP Class 2 (n = 42)	*P* value
Gender			0.009
Male	65 (55%)	33 (79%)	
Female	52 (45%)	9 (21%)	
Age	55.8 (SD^b^ =14.5)	66.0 (SD = 13.9)	0.0001
Site			0.5507
Extremity	48 (41%)	17 (40%)	
Head & Neck	17 (15%)	9 (21%)	
Trunk	52 (44%)	16 (38%)	
Ulceration			< 0.0001
No	107 (91%)	14 (33%)	
Yes	10 (9%)	28 (67%)	
Breslow thickness	1.4 (SD = 1.1)	3.7 (SD = 2.7)	< 0.0001
T stage			< 0.0001
T1	50 (43%)	1(2%)	
T2	51 (44%)	11 (26%)	
T3	13 (10%)	14 (33%)	
T4	3 (3%)	16 (38%)	
SNB^c^ result			0.0105
Positive	10 (9%)	10 (24%)	
Negative	107 (91%)	32 (76%)	
AJCC^d^ stage			< 0.0001
Stage I	91 (78%)	5 (12%)	
Stage II	16 (13%)	24 (57%)	
Stage III	10 (9%)	13 (31%)	

a gene expression profile

b standard deviation

c sentinel node biopsy

d American Joint Committee on Cancer

Within the stage III cohort, 11 patients received adjuvant high‐dose interferon‐alfa 2b or pegylated interferon‐alfa 2b. Two stage III patients enrolled in clinical trials. Ten stage III patients opted for expectant observation. With a median follow‐up time of 44.9 months for event‐free cases, 29 patients experienced recurrence with a median time to recurrence of 13.3 months (range: 1.6‐51.4 months). Of the 29 patients with recurrence, 10 (34%) were SNB‐positive and 19 (66%) were SNB‐negative (Table 2). For GEP, 23 (79%) patients who had recurrence were GEP Class 2, while 6 (21%) were Class 1. Of the 19 node‐negative patients who experienced recurrence, 14 (74%) were Class 2. Nine of 10 patients with a positive sentinel node and GEP Class 2 melanoma recurred. Eleven of 13 patients with AJCC stage III and GEP Class 2 melanoma recurred, including 7 patients who received adjuvant therapy (Table 3). The vast majority (9 of 11, 82%) of first recurrences in the AJCC stage III/GEP Class 2 cohort were to distant visceral sites. At the time of last follow‐up, 9 patients in the GEP Class 2 group had expired due to any cause compared to one in the GEP Class 1 group.

**Table 2:** Recurrence rates with GEP^a^ and SNB^b^

	Recurrence‐free n (% of row)	With recurrence n (% of row)
Class 1 (n = 117)	111 (95%)	6 (5%)
Class 2 (n = 42)	19 (45%)	23 (55%)
SNB‐negative (n = 139)	122 (88%)	19 (14%)
SNB‐positive (n = 20)	10 (50%)	10 (50%)
Class 1/SNB‐negative (n = 107)	102 (95%)	5 (5%)
Class 1/SNB‐positive (n = 10)	9 (90%)	1 (10%)
Class 2/SNB‐negative (n = 32)	18 (56%)	14 (44%)
Class 2/SNB‐positive (n = 10)	1 (10%)	9 (90%)

a gene expression profile

b sentinel node biopsy

**Table 3:** Sites of recurrence according to AJCC^a^ stage and GEP^b^ class

AJCC stage	GEP class (n)	Recurrence‐free	With recurrence n, site of first recurrence; second recurrence
Stage I	Class 1 (91)	88	1 in transit 2 nodal
	Class 2 (5)	5	0
Stage II	Class 1 (16)	14	1 subcutaneous 1 in transit
	Class 2 (24)	12	1 local subcutaneous 1 subcutaneous 1 local; lung 3 in transit 1 nodal; bone 1 nodal; brain 1 lung/adrenal/subcutaneous 3 lung
Stage III	Class 1 (10)	9	1 in transit/liver
	Class 2 (13)	2	1 subcutaneous; brain 2 in transit 1 in transit; small bowel/liver 1 in transit; liver 2 brain 1 lung 1 lung/liver 1 lung/subcutaneous 1 liver

a American Joint Committee on Cancer

b gene expression profile

### 3.2 Recurrence‐free and distant‐metastasis‐free survival analysis

**Figure 1:**
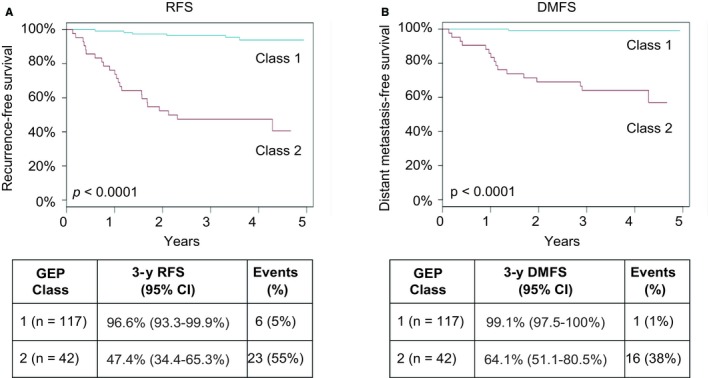
Kaplan‐Meier analysis of recurrence‐free survival (RFS; A) and distant metastasis‐free survival (DMFS; B) by gene expression profiling (GEP) class in the prospective cohort (n = 159). *P*‐values were calculated by log rank test. Tables report number of patients for each GEP class, 3‐yr survival rates with 95% confidence intervals, the number of overall events for a given outcome and class, and percentages of the class experiencing the event

On univariate analysis, Breslow thickness, ulceration, SNB result, and GEP class were significantly associated with RFS and DMFS (*P* < 0.001 for all variables; Tables 4 and 5), while age was only significant for RFS (*P* = 0.0113). Tumor location and gender were not statistically significant for either outcome in univariate analysis (*P* > 0.05), and thus, along with age for RFS, were not included in subsequent multivariate analysis. In multivariate analysis, the hazard ratios (HR) for GEP Class 2 were 9.2 (*P* < 0.001, 95% confidence interval (CI) = 3.0‐28.5) and 19 (*P* < 0.01, 95% CI = 2.12‐170.5) for RFS and DMFS. SNB result was also associated with RFS and DMFS (*P < *0.02, HR = 3.5, 3.7, 95% CI = 1.4‐9.1, 1.2‐11.3, respectively). Breslow thickness was not nominally significant for DMFS in multivariate analyses (*P* = 0.06, HR = 1.2, 95% CI = 1.0‐1.4) but was statistically significant for RFS (*P* = 0.015, HR = 1.15, 95% CI = 1.01‐1.31). Ulceration was not found to be significant for either endpoint in multivariate analyses. For GEP Class 1 patients, the observed 3‐year RFS (Figure 1A) and DMFS (Figure 1B) rates were 96.6% and 99.1%, respectively, compared to 47.4% and 64.1%, respectively, for GEP Class 2 patients (*P* < 0.0001 for Class 1 vs Class 2 for both endpoints). GEP subclass provided additional stratification with 3‐year RFS and DMFS rates for subclass 2B cases of 39.5% and 59.6% (Figure S1).

**Table 4:** Recurrence‐free survival analysis

	Univariate analysis	Multivariate analysis
Age	*P* = 0.011 HR^a^ 1.0; 95% CI^b^ 1.0‐1.1	*P* = 0.14 HR 1.0; 95% CI 0.99‐1.1
Breslow thickness	*P* < 0.0001 HR 1.4; 95% CI 1.3‐1.5	*P* = 0.015 HR 1.2; 95% CI 1.0‐1.3
Ulceration	*P* < 0.0001 HR 5.4; 95% CI 2.6‐11.4	*P* = 0.69 HR 0.8; 95% CI 0.3‐2.1
SNB^c^ results	*P* < 0.0001 HR 5.1; 95% CI 2.4‐11.1	*P* = 0.008 HR 3.5; 95% CI 1.4‐9.1
GEP^d^ class	*P* < 0.0001; HR 15.0; 95% CI 6.1‐37.0	*P* = 0.0001 HR 9.2; 95% CI 3.0‐28.5

a hazard ratio

b confidence interval

c sentinel node biopsy

d gene expression profile

**Table 5:** Distant metastasis‐free survival

	Univariate analysis	Multivariate analysis
Breslow thickness	*P* < 0.0001 HR^a^ 1.5; 95% CI^b^ 1.3‐1.7	*P* = 0. 06 HR 1.2; 95% CI 1.0‐1.4
Ulceration	*P* < 0.0001 HR 12.6; 95% CI 4.0‐38.9	*P* = 0.14 HR 2.5; 95% CI 0.7‐8.5
SNB^c^ results	*P* < 0.0001 HR 7.8; 95% CI 3.0‐20.3	*P* = 0. 019 HR 3.75; 95% CI 1.2‐11.3
GEP^d^ class	*P* = 0.0001; HR 55.1; 95% CI 7.3‐415.9	*P* = 0.009 HR 19.0; 95% CI 2.1‐170.5

a hazard ratio

b confidence interval

c sentinel node biopsy

d gene expression profile

## 4 DISCUSSION

GEP molecular class was an independent prognostic variable for disease recurrence in this single‐institution prospective study. SNB result was also an independent prognostic variable for disease recurrence. GEP class was significantly associated with but independent from other clinical and histopathological prognostic variables such as gender, age, Breslow thickness, and SNB status. Of the 10 deaths from all causes observed in the current study, 90% occurred in patients with a GEP Class 2 tumor, which is in agreement with prior publications.^5^, ^8^

The current study has a median follow‐up duration of 3.5 years overall and 3.7 years for patients without a recurrence. The median time to recurrence in this cohort was 13.3 months (1.1 years) and 90% of recurrences occurred by 30.1 months (2.5 years), indicating follow‐up duration was adequate to confirm the accuracy of the GEP test and that surveillance imaging for early identification of metastatic disease can be performed in that time period. Although melanoma recurrence can occur decades after initial diagnosis,^11^ the time duration between initial diagnosis and disease recurrence, i.e. disease‐free interval, has been shown to be prognostic of melanoma patient outcome.^12^, ^13^, ^14^, ^15^ Patients with early recurrence likely harbor tumors with more aggressive phenotype than those with delayed recurrence. In addition, low tumor burden has been demonstrated to be a robust predictor of outcome for surgical management of metastasis and for novel immune checkpoint therapies.^16^ Thus, identification of patients with early recurrence and development of effective adjuvant therapy for these patients would likely have significant impact on overall melanoma patient outcomes.

Both GEP class and SNB positivity were independently prognostic of disease recurrence in this prospective study. The combination of GEP test and SNB may improve our ability to determine prognosis in patients with primary CM, as has been previously demonstrated.^6^, ^10^ Gerami et al reported that the combination of SNB and GEP testing indeed allowed for separation of primary melanoma patients into distinct subgroups with low, moderate, and high risk for recurrence.^6^ Zager et al reported similar results in an independent cohort of 523 patients.^10^ Thus, use of both SLNB and GEP testing in the staging work‐up of primary melanoma patients may allow separation of groups at very low risk, moderate risk, and very high risk for recurrence.

Two recent large retrospective analyses have shown the additive prognostic value of GEP testing in early stage melanoma patients especially in stage II melanoma patients.^9^, ^10^ While Zager et al did not observe a statistical significance in DMFS for stage I subgroup (n = 264), Gastman et al did show a statistical significance in DMFS with a larger stage I cohort (n = 333). Due to the small sample size in this prospective cohort, further analysis specific to stage subgroups or age subgroups would not likely yield meaningful results. With the coming maturation of the large multicenter prospective study of GEP testing in primary melanoma patients,^8^ further subgroup analysis of this type may yield more information regarding the subgroup‐specific utility of GEP testing.

Recently, other molecular testing approaches for prognostication of primary melanoma have been proposed. Nsengimana et al performed an independent validation of a whole‐genome mRNA profiling classification.^17^, ^18^ Archival tumor tissues from 300 patients (224 primary and 76 metastatic) were evaluated. Gene signature classification was significantly correlated with Breslow thickness, ulceration, mitotic rate, and melanoma‐specific survival. Other gene expression profiles for melanoma have been described and reported additive prognostic values in addition to clinical and histological factors.^19^, ^20^ MicroRNA‐21, ‐137, and ‐203 have been evaluated in primary melanoma tissues and found to be independently associated with survival.^21^, ^22^, ^23^ Down‐regulation of miRNA‐150‐5p and miRNA142‐3p/142‐5p duplex correlated with poor survival in metastatic melanoma patients.^24^ However, no prospective validations of these approaches have been performed.

Following the validation of several efficacious agents against metastatic melanoma,^25^, ^26^, ^27^, ^28^, ^29^ therapies are now been applied in the adjuvant setting. Over the last 2 decades, four agents have been approved for adjuvant use in stage III melanoma: interferon, ipilimumab, nivolumab, and the combination of dabrafenib and trateminib.^30^, ^31^, ^32^, ^33^ Pembrolizumab has also shown efficacy in stage III patients.^34^ While only high‐dose interferon is approved for use in T4 Stage II melanoma patients, clinical trials testing PD‐1 inhibitors in stage IIB‐IIC patients are currently ongoing. In light of these trials, identification of a subset of high risk Stage II melanoma would allow for rational clinical trial design and expedient determination of the clinical efficacy of adjuvant targeted and immunotherapies. Furthermore, the observation that 11 of 13 Stage III GEP Class 2 patients recurred within 2 years suggests these patients could have harbored occult disease at the time of primary melanoma presentation and should be treated aggressively. Of interest, 28 patients in this study had both GEP testing and BRAF mutation status analyzed. An association between the two molecular markers was not observed for these patients, as BRAF was mutated in only three of the patients—one Class 1 and two Class 2 (Fisher's Exact *P* = 0.59). GEP testing can identify melanoma patients at high risk for recurrence, however clinical trials are needed for these high‐risk patients to determine the optimal treatment strategy, in particular for adjuvant therapy decision making.

The results from this single‐center prospective study confirm prior retrospective and prospective studies in support of the use of GEP testing in combination with current staging procedures for prognostication of CM. While prior clinical utility studies have been published to indicate current clinical applications of this test for patient management,^35^, ^36^, ^37^, ^38^, ^39^ the ongoing expansion of adjuvant therapy choices for CM patients supports the need for accurate risk assessment in patients eligible for adjuvant therapy and suggests additional potential utility for this prognostic tool.

## DISCLOSURES

ECH is on the speaker bureaus of Amgen, Inc and Castle Biosciences, Inc

## AUTHOR CONTRIBUTIONS

Study conception and design: Hsueh, Schwartz, Hurley, Patel. Acquisition of data: Keller, Chang, Patel, Lizalek, Hsueh. Analysis and interpretation of data: Hsueh, Keller, Schwartz. Drafting of manuscript: Keller, Schwartz, Chang, Hurley, Hsueh. Critical revision: Keller, Schwartz, Hsueh.
