# Supplementary figures and images for: Prospective validation of the prognostic 31‐gene expression profiling test in primary cutaneous melanoma
Source: Cancer Med. 2019 Apr 5;8(5):2205–12. doi: 10.1002/cam4.2128 (PMC6536922; doi:10.1002/cam4.2128)

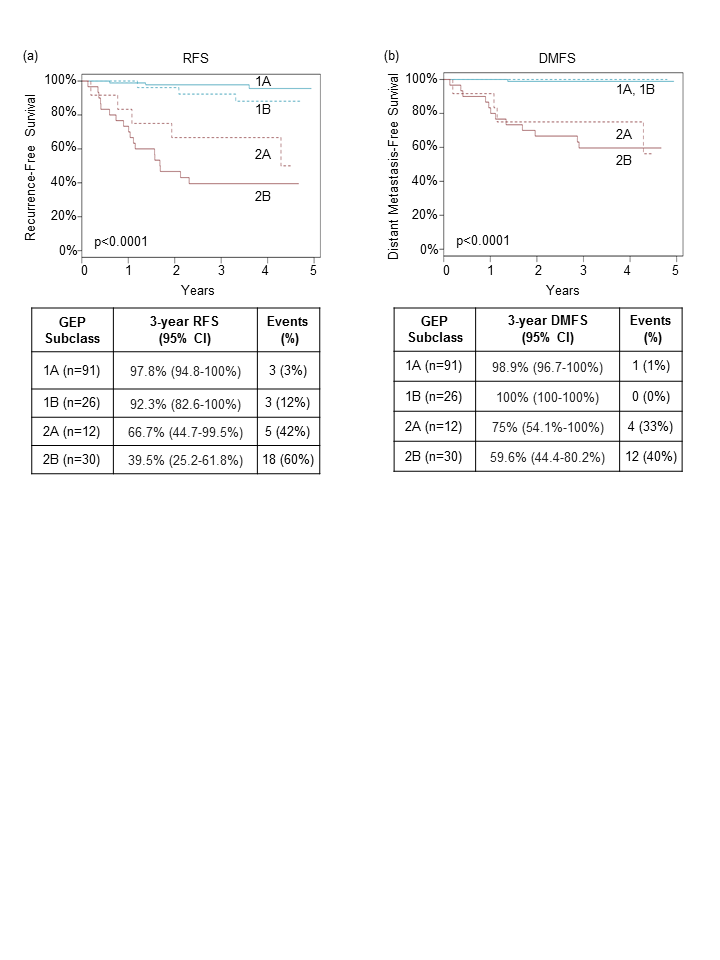

Supplement: Supplementary file 1 [file CAM4-8-2205-s001.tif]
